# Tracking progress toward a climate-friendly public food service strategy: assessing nutritional quality and carbon footprint changes in childcare centers

**DOI:** 10.1186/s12937-024-00917-5

**Published:** 2024-01-27

**Authors:** Matilda Nordman, Anne Dahl Lassen, Lene Møller Christensen, Ellen Trolle

**Affiliations:** National Food Institute, Technical University of Denmark, Kemitorvet 201, 2800 Kgs., Lyngby, Denmark

**Keywords:** Public food procurement, Public food service, Sustainable diets, Childcare, Carbon footprint

## Abstract

**Background:**

Public food procurement and catering are recognized as important leverage points in promoting sustainable and healthy dietary habits. This study aimed to analyze changes in nutritional quality and carbon footprint (CF) of food service in childcare centers in the City of Copenhagen from 2018 to 2022, following a new climate-friendly food strategy in 2019. The strategy has a target of decreasing the CF of municipal food service by 25% before 2025 compared to a 2018 baseline.

**Methods:**

Key initiatives in the municipality’s strategy included creating guidelines for food-service providers to reduce their CF while ensuring meal nutritional quality and providing food professionals an advisory process to develop necessary competencies.

In this quasi-experimental study, food procurement data from Copenhagen’s childcare centers (*n* = 356 [2022]) from 2018 and 2022 were combined with CF and nutrient composition data. Dietary CF and food and nutrient content were calculated per 10 MJ of energy and compared to guideline targets. Furthermore, data for 2022 were analyzed separately for institutions that had received an advisory process (*n* = 87) and those that had not yet (*n* = 269).

**Results:**

On average, the CF of the food procurement decreased by 15%, mainly driven by a decrease in ruminant meat purchases (-37%). While the procurement of plant-based protein sources (pulses, nuts, seeds) increased by 25%, it was still considerably below targets. Nutrient content did not substantially change, and recommendations for calcium, iron, vitamin D, sodium, and total and saturated fat were not met in either measurement year. Institutions that had received an advisory process had a 14% lower CF in 2022 than institutions that had not.

**Conclusions:**

With the observed 15% CF reduction, Copenhagen’s childcare centers are on track to reach the 25% reduction goal outlined in the municipality’s food strategy by 2025. Nutritional quality was largely unchanged, but further efforts to increase especially the consumption of plant-based protein sources, while simultaneously reducing meat and animal-based fat, and maintaining sufficient dairy consumption, are needed to improve nutritional quality and reach the target CF reduction in the coming years. Providing training for food professionals could play an important role in seeing the changes through.

## Introduction

The global food system is a leading driver of environmental degradation through its contributions to anthropogenic greenhouse gas emissions (GHGE), land system change, fresh-water withdrawal, biodiversity loss, and eutrophication [1]. Public food procurement is recognized as an important leverage point in shifting the food system towards sustainable practices and promoting healthy dietary habits [2]. With its large buying power, public food procurement has the potential to influence both consumption and production of food to deliver benefits on multiple levels and act as a trendsetter by shaping societal food norms [3, 4]. Target 12.7 of the UN Sustainable Development Goals explicitly focuses on “promoting public procurement practices that are sustainable in accordance with national policies and priorities”. Despite the increasing recognition and potential of public food procurement, it remains an underexplored topic, as highlighted by Swensson et al. [3].

Public food procurement and catering encompass the purchase and provision of food in various public settings and government institutions, including educational institutions, such as schools and childcare centers, as well as public hospitals, government canteens, care homes, etc. The responsibility for public food procurement may vary across countries, occurring at the state, regional, or municipal levels, and can be supported by different regulatory frameworks [5].

In Copenhagen, the capital city of Denmark, the municipal food service provides citizens with more than 70 000 meals every day, mainly across childcare centers, schools, and care homes. In 2019, the City of Copenhagen adopted a new food strategy, which outlines the goal of reducing the carbon footprint (CF) of public food service in the municipality by 25% before 2025 compared to a 2018 baseline while simultaneously ensuring nutritional and culinary quality and a high proportion of organic food procurement [6]. At the same time, Copenhagen became a signatory of the Cool Food Pledge (CFP). The CFP is a global initiative by the World Resources Institute (WRI), aiming to help food providers set science-based targets for reducing their food-related CF and track progress toward fulfilling them [7]. To align with the Paris climate agreement, CFP signatories should aim for a 25% absolute reduction in food-related GHGE by 2030 [7]. An increasing number of initiatives toward environmentally sustainable public food practices are also seen elsewhere. E.g., in 2022, in the German city of Freiburg, the city’s council decided that only vegetarian meals should be served in primary schools and daycare centers [8]. This decision has sparked controversy about concerns for proper child nutrition [9].

In promoting environmental sustainability in food service, focus on nutrition and health is important, especially in nutritionally vulnerable groups such as young children. Monitoring diet composition is important to ensure that the nutritional quality of meals is not compromised as the sustainability agenda is promoted. Previous studies on sustainability aspects in public meals and procurement have mainly focused on school meals [4, 10–16], while few have looked at the early childcare setting. Although younger children, due to lower energy intake, have a relatively small dietary CF [17], food preferences and dietary habits are established early in life and have the potential to carry over into adulthood [18]. Therefore, establishing healthy and sustainable eating habits early in life is important, and public meals play a role in promoting healthy dietary habits [19]. In young children, meals consumed in childcare make a significant contribution to children’s total weekday food and nutrient intake [20].

To date, few studies have focused on quantitatively evaluating both environmental and nutritional outcomes of public food service strategies and policies in wide-scale real-world settings. To enable informed decision-making and policy formulation for public food procurement and service, monitoring of environmental impacts as well as nutritional quality in these settings is crucial. Further studies on the potential positive and negative effects of sustainable public procurement have been called for, as well as monitoring and evaluation of health and sustainability food service guidelines [21, 22].

The objective of this study was to assess the changes in nutritional quality and CF of public food service in child-care centers in Copenhagen municipality between 2018 and 2022 to examine the consequences of a 2019 political strategy aimed at reducing the CF in public food service in the municipality.

## Methods

### Study design

This study was an uncontrolled before and after study of food procurement in child-care centers in the City of Copenhagen. By analyzing temporal trends in food procurement data from 2018 to 2022, this study represents a mid-way evaluation in the implementation of the new food strategy introduced in 2019. Besides previously bringing forth the scientific basis for the development of guidelines for healthy and climate-friendly meals in child-care centers [23], and carrying out the present mid-way analyses, the researchers’ role in the project (the formulation of the strategy and its implementation) was purely observational.

#### Copenhagen’s 2020–2025 Food Strategy

**Fig. 1:**
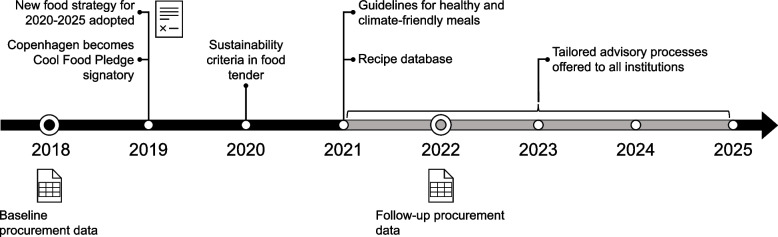
A timeline of the City of Copenhagen’s food strategy for 2020–2025: key initiatives and data collection points

An overview of different key initiatives that were part of the municipality’s food strategy is provided in Fig. 1, with emphasis on initiatives relevant to CF reduction. An important part of the strategy was the creation and provision of guidelines for food-service providers on how to achieve CF reduction while ensuring the nutritional quality of the meals served [24]. The target group-specific guidelines for meeting the 25% CF reduction goal were created based on scenario analyses of the municipality’s 2018 food procurement data [23, 25], and are in accordance with the Danish food-based dietary guidelines, which take departure in the EAT Lancet reference diet for a global healthy and sustainable diet [26]. The guidelines outline detailed amounts of different foods to be served, as well as suggested serving frequencies [23]. The guidelines were distributed to all childcare centers in the municipality in November 2021, along with an extensive recipe database supporting the implementation of healthy and climate-friendly meals [27]. In addition, a central part of the implementation of the strategy is an ongoing comprehensive upskilling effort among the municipality’s food professionals, running from 2021 to 2025. Personnel across all child-care centers in the municipality are offered a tailored advisory process lasting 3, 6, or 12 months. The advisory process is facilitated by food consultants and is complemented by courses and workshops to incorporate healthy and climate-friendly meal practices. Finally, to encourage the procurement of foods with central importance to the strategy implementation and provide competitive pricing, additional sustainability criteria were added to the municipality’s food tender in 2020 [28].

Additional initiatives focusing on food waste reduction and food communities took place but are not described in the present paper.

### Data sources and compilation

**Fig. 2:**
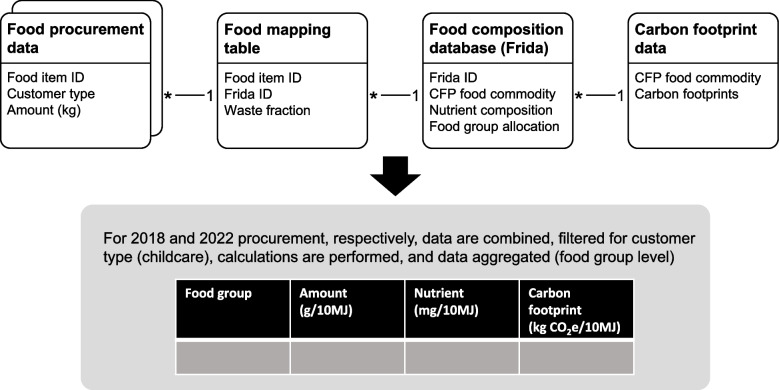
Simplified overview of data sources and data compilation for analyses. CFP: Cool food Pledge [7]

Data from three separate sources formed the basis for analyses in the present study: (1) food procurement data for the City of Copenhagen in 2018 and 2022, (2) the Danish food composition database, and (3) CF metrics for different food commodities. Figure 2 provides a simplified overview of the compilation of data for analysis. In order to combine the food procurement data with food composition data, a food mapping table was created, where each unique item in the food procurement data was mapped to the best-fitting item in the food composition database. In addition, waste factors were assigned to the unique food items in the food mapping table to account for the inedible parts of foods in retail form (i.e., peels, skin and bones, etc.). The waste factors assigned to different foods are described in the supplementary material of Lassen et al. [23]. Data compilation and analyses were carried out in Microsoft Excel and the tidyverse package family within R statistical software.

#### Food procurement data

Food procurement data was provided by the City of Copenhagen for the years 2018 and 2022 for all target groups of public food service in the municipality. The data covered nearly the entirety of food purchased by municipal institutions and included a main wholesale food supplier as well as other smaller suppliers. Based on overall food expenses in the municipality, an estimated 70–80% of all food was purchased from one main wholesale food supplier in 2018 and 2022. In addition to the main food supplier, other suppliers providing smaller quantities of single food categories (e.g. fish) were included in the analysis data to ensure data representativeness across food groups. Suppliers that provided small quantities of a variety of foods were not included for simplicity. Missing procurement weights from smaller suppliers were estimated from procurement expenses based on food-group-specific kilo prices. For the 2018 dataset, it was estimated that the final dataset for analysis represented approximately 80% of the total food procurement in the municipality, with good representativeness across food groups [23]. The same procedure for the selection of supplier data was applied for 2022 data in dialogue with food consultants knowledgeable about potential changes in procurement patterns. Therefore, representativeness is estimated to be similar for 2022 data (approximately 82% based on total food expenditure), with good comparability across measurement years.

The food procurement dataset contained information on the food items purchased, the amount, and customer information, which allowed for coding customers according to customer type, e.g., childcare center, school, care home, etc.

#### Food composition data and carbon footprint data

Information on the nutritional content of foods was obtained from the Danish Food Composition Database Frida version 3, complemented with newer data for selected food products (salmon, several cereals, seeds, and nuts) [29]. The food composition table was checked for missing values on added sugar and micronutrients and complemented with values from the Swedish food composition table [30] or estimated from similar products, where appropriate.

CFs of different foods were obtained from the WRI Cool Food Pledge (CFP) calculator (version June 2022) [7]. The CFP calculator contains region-specific food-related CFs for a total of 57 food commodities: the GHGE from cradle to retail gate represented as kg CO_2_e per kg of bone-free food product (retail weight). The metrics used in the present study were the European average values for GHGE from agricultural supply chains (CFP Metric 2), originating from Poore and Nemecek [31], global average values for carbon opportunity costs (CFP Metric 4) from Searchinger et al. [32], as well as the sum of Metric 2 and 4 (total food related carbon footprint). While Metric 2 represents the production-related emissions, Metric 4 translates global agricultural land use into GHGE by estimating the amount of carbon lost from plants and soils as a result of agricultural expansion due to additional production of a food [7]. The food items in the food composition database were matched with the food commodities in the CFP calculator, as previously described in detail elsewhere [23].

In some cases, the food items in the food procurement data represented composite foods (e.g., spring rolls and meatballs), which were not possible to map directly to an item in the food composition table or a CFP food commodity. For these items, the nutrient composition and CF were calculated using a standard recipe, approximating the proportion of different ingredients in the composite food and thereby estimating its nutrient composition and CF.

Based on nutritional, environmental, and functional characteristics (e.g. dry vs. fresh fruit), food items in the food composition database were categorized into main food groups and food sub-groups. These are described in more detail in the supplementary material of Lassen et al. [23].

### Evaluation of nutritional quality and carbon footprint

For the 2018 and 2022 procurement, respectively, data were combined, and calculations were performed on the pooled data for all childcare centers. Calculations per person and day were not possible; therefore, to enable comparison of nutritional quality and CFs with recommendations and across the different measurement years, procurement amounts (kg) in each year were proportionally adjusted to a total energy level of 10 MJ (2390 kcal). This is the same energy level to which the reference diet, underlying the guidelines for healthy and climate-friendly diets, was previously modelled [23]. Calculations of nutrient content and CF were carried out on the edible fraction of purchased foods on food item level (Frida ID) and results were subsequently aggregated to food group and whole diet level.

The procurement data of childcare centers cover meals for children aged 1–6 years, but primarily 2–5 years. Nutritional quality was assessed by comparing the nutrient density of the procured foods to nutrition recommendations for 2–5-year-olds from the Nordic Nutrition Recommendations 2012. Recommended daily intakes of nutrients were upscaled to recommendations per 10 MJ using the age group-specific reference energy requirement of 5.3 MJ/day [33]. In addition, purchased food amounts (edible fraction) were compared to target amounts in the modeled scenario diet which forms the basis for guidelines for healthy and climate-friendly diets in childcare centers [23]. The meals represented in the food procurement and the guidelines are breakfast (for a small proportion of children), lunch, and 1–2 in-between meals. Previous estimates indicate that childcare centers provide up to 70% of children’s daily energy needs [34]. Therefore, it was assumed that the nutrient density for this mix of main and in-between meals is comparable to that of a whole day, and consequently, recommended nutrient density for a whole diet was used to evaluate nutrient adequacy.

### Evaluation of the role of the advisory process

To estimate the potential impact of institutions having received an advisory process, analyses of 2022 data were carried out separately for those childcare centers that had received an advisory process (*n* = 87) and those that had not yet (*n* = 269). Institutions having completed the entirety of their advisory processes in 2021 or 2022, or started a process within the first quarter of 2022 but not finished it by the end of the year, were considered as having received an advisory process, while remaining institutions were considered as not having received an advisory process. The analysis excluded fish procurement from one supplier for which data was not available on customer level, representing 0.06% of total procurement weight in childcare centers.

## Results

### Background information

In the final food procurement datasets, the childcare sector represented approximately one-third of the total food expenditure in Copenhagen in 2018 and 2022, making young children the largest target group within public food service in the municipality. The datasets included all municipally owned childcare centers (*n* = 356 [2022]), whose total food purchases amounted to roughly 3 million kg in both years. In the final datasets, more than 99% of all food in childcare centers was purchased from the main wholesale supplier, with the remaining fraction representing fish purchased from other suppliers.

### Food content

The amounts of different foods procured (edible fraction, g/10 MJ) by childcare centers in Copenhagen in 2018 and 2022 are displayed in Table 1, with a comparison to target amounts based on the modeled diet foundational to the guidelines for healthy and climate-friendly meals in childcare centers [23]. The most prominent changes in procurement patterns from 2018 to 2022 were decreases in the amounts of total meat (-22%), especially ruminant meat (-37%) and pork (-30%), milk (-17%), and relative increases in the purchase of eggs (+ 34%), pulses (+ 41%), processed plant-based protein-rich foods (+ 250%), and plant-based dairy alternatives (+ 250%). There was a shift in the usage of fat away from animal-based fats (-20%) to the use of more plant-based fats (+ 28%). Likewise, the energy contribution from animal-based foods to total energy was decreased from 30 E% in 2018 to 26 E% in 2022, while the contribution of protein from animal-based foods was decreased from 50 to 46% of total protein (excluding negligible energy and protein contributions from mixed composite food groups, data not shown).

**Table 1:** Food procurement in childcare centers (edible fraction, g per 10 MJ) in 2018 and 2022, the percent change in purchased amounts, and the target amounts from guidelines for healthy and climate-friendly meals in childcare centers based on modeled diet [23]

**Food group**	**Procured amount (g/10 MJ)**			**Target (g/10 MJ)**
	**2018**	**2022**	**% Change**	
Bread and cereals^a^	336	328	-2%	280
Whole grain, g ingredient	126	114	-9%	
Potatoes	100	95	-5%	100
Vegetables total^b^	238	258	8%	278
Fruit total^c^	182	185	2%	277
Pulses, dry^d^	8.4	11.8	41%	30
Processed plant-based protein-rich foods^e^	0.7	2.6	250%	-
Tree and ground nuts	1.3	1.4	10%	15
Seeds^f^	4.0	3.8	-5%	9
Milk	308	256	-17%	364
Dairy foods^g^	54	53	-3%	36
Cheese	19	20	5%	20
Plant-based dairy alternatives	3.8	8.8	129%	-
Meat total^h^	71	56	-22%	47
Beef and lamb	25	16	-37%	9.5
Pork	16	11	-30%	9.5
Poultry	30	28	-5%	28
Egg	16	21	34%	23
Fish, total^h^	52	53	2%	63
Fats, plant-based^i^	37	47	28%	46
Fats, animal-based^i^	28	23	-20%	4
Coffee and tea	3	3	16%	3
Discretionary foods and beverages	26	28	9%	26
Condiments and seasoning	15	16	10%	15

^a^Combination of grains/flour and bread; ^b^Includes mushrooms; ^c^Includes berries, dried fruit, and a limited amount of fruit juice; ^d^Pulses purchased as a mix of cooked and dry pulses but are here expressed as dry weight ^e^Contains different soy-, pea-, and mycoprotein-based products (tofu, patties etc.).; ^f^Does not include seeds in bread; ^g^All other dairy products except milk, cheese and butter. The target for the sum of milk and dairy foods should be 400 g/10 MJ; ^h^Meat and fish are predominantly raw but also contain limited amounts of processed products; ^i^Includes fat-based products, e.g., sauces and dressings

Compared to the modeled diet that is foundational to the guidelines for healthy and sustainable meals for childcare centers [23], the procurement of plant-based protein sources (pulses, nuts, and seeds) in 2022 was one-third of the target (in total, 54 g target vs 17 g procured per 10 MJ), while meat and animal-based fats were above targets. In addition, the sum of vegetables and fruit (443 g/10 MJ) was lower than the guideline target (555 g/10 MJ). The sum of milk and other dairy foods in 2022 was also lower than target (309 vs. 400 g/10 MJ), representing a decrease since 2018 driven especially by a decrease in milk.

### Nutrient content

The nutrient content of the food procurement was largely unchanged from 2018 to 2022, and for the most part, only minor changes can be observed (Table 2). The energy contributions from different macronutrients were within acceptable ranges for individuals, but when compared to narrower targets for dietary planning purposes, energy contribution from fat was slightly too high and protein slightly too low (13 E% corresponding to 79 and 76 g protein/10 MJ in 2018 and 2022, respectively). The contents of saturated fat and sodium were higher than recommended in both measurement years, while iron, calcium, and vitamin D were below recommendations. Lassen et al. have previously estimated that 136 mg of calcium could be added per 1.13 l of drinking water in the diet [23], but the total content of calcium would still be below recommendation: 1002 mg and 963 mg/10 MJ in 2018 and 2022, respectively. Potassium was borderline below recommendation in 2022.

**Table 2:** Content of nutrients in food procurement in childcare centers (per 10 MJ) in 2018 and 2022, and comparison to recommendations. Nutrients not fulfilling recommendations are marked in bold

**Nutrient**^**a**^	**Nutrient content per 10 MJ**		**% Change**	**Recommendation**^**b**^
	**2018**	**2022**		
Protein, total, E%	**13**	**13**	-3%	10–20 (15)
Carbohydrates, E%	52	52	-1%	45–60 (52–53)
Added sugar, E%	3	3	6%	≤ 10
Fat, total, E%	**34**	**35**	2%	25–40 (32–33)
Saturated fatty acids, E%	**12**	**12**	-1%	≤ 10
n-3 fatty acids, E%	1.3	1.4	6%	≥ 1
Dietary fiber, g	39	39	-1%	≥ 20
Vitamin A, RE	1263	1161	-8%	≥ 660
Vitamin D, µg	**3**	**3**	-7%	≥ 19
Vitamin E, α -TE	13	16	18%	≥ 9
Thiamine, mg	1.5	1.5	-3%	≥ 1.1
Riboflavin, mg	1.6	1.5	-6%	≥ 1.3
Niacin, NE	26	24	-6%	≥ 17
Vitamin B6, mg	1.9	1.8	-2%	≥ 1.3
Folate, µg	489	491	0%	≥ 151
Vitamin B12, µg	6.3	5.3	-16%	≥ 1.5
Vitamin C, mg	140	151	8%	≥ 57
Sodium, mg	**2636**	**2736**	4%	≤ 2400^c^
Potassium, mg	3447	**3385**	-2%	≥ 3396
Calcium, mg^d^	**866**	**827**	-5%	≥ 1132
Magnesium, mg	375	372	-1%	≥ 226
Phosphorus, mg	1617	1555	-4%	≥ 887
Iron, mg	**13**	**13**	0%	≥ 15
Zinc, mg	11	11	-5%	≥ 11
Iodine, µg	171	171	0%	≥ 170
Selenium, µg	49	48	-2%	≥ 47

^a^Bioavailability and losses of vitamins and minerals from cooking not accounted for. ^b^Based on recommended nutrient density per 10 MJ for dietary planning for 2–5-year-olds from the Nordic Nutrition Recommendations 2012. Macronutrient recommendations are expressed as acceptable ranges for individuals and narrower ranges for planning purposes in parentheses. ^c^Based on recommendation for adults. ^d^An estimated 136 mg of calcium per 10 MJ can be added from 1.13 l of water, bringing the total amount of calcium to 1002 and 963 mg/10 MJ in 2018 and 2022, respectively

### Carbon footprint

**Fig. 3:**
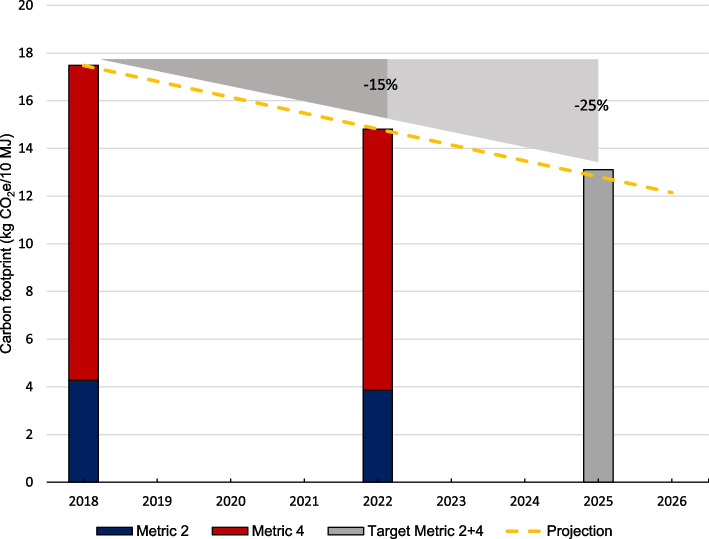
The carbon footprint of food procurement in 2018 and 2022 as measured by Cool Food Pledge Metric 2 and Metric 4 [7], and the target for the total food-related carbon footprint in 2025 following the municipality’s strategic goal [6]. The yellow line shows the projected reductions if a continued linear reduction is assumed beyond 2022

From 2018 to 2022, the food-related CF reduced by 10% and 15%, as measured by CFP Metric 2 and Metric 2 + 4, respectively. The emissions from agricultural supply chains (Metric 2) measured 4.3 and 3.9 kg CO_2_e/10 MJ in 2018 and 2022, respectively, while corresponding numbers were 17.5 and 14.8 kg CO_2_e/10 MJ when including carbon opportunity costs (Metric 2 + 4). If a linear reduction in the total food-related CF (Metric 2 + 4) is assumed beyond 2022, the childcare centers are on track to meet the municipality’s 25% reduction target by 2025 (Fig. 3).

The largest CF reduction came from the reduced amount of ruminant meat, contributing to a reduction of 2.3 kg CO_2_e of the total 2.7 kg CO_2_e reduction per 10 MJ (Metric 2 + 4, data not shown). Notable reductions were also seen from decreased amounts of milk (-0.4 kg CO_2_e/10 MJ) and animal-based fats (-0.3 kg CO_2_e/10 MJ) from 2018 to 2022.

### The role of the advisory process

Full results from separate analyses on institutions that had and had not gone through an advisory process are displayed in Table 3 and Table 4. Results indicated that institutions that had gone through an advisory process had a 14% lower CF (Metric 2 + 4) than institutions that had not gone through an advisory process (Table 4). This puts the recipients of an advisory process at a CF reduction of 24% from the common baseline of all childcare centers in 2018 and non-recipients at a reduction of 12%. Institutions that had gone through an advisory process procured less total meat (-19%), especially ruminant meat (-45%), and animal-based fats (-37%) and more plant-based protein sources: pluses (+ 22%), nuts (+ 59%), and seeds (+ 33%) and plant-based fats (+ 18%), compared to institutions that had not gone through an advisory process (Table 3). Nutrient content and adequacy were not markedly different (Table 4).

**Table 3:** Food procurement in 2022 for childcare centers who have and have not received advisory process (edible fraction, g per 10 MJ), the percent difference between groups, and the target amounts from guidelines for healthy and climate-friendly meals in childcare centers based on modeled diet [23]

**Food group**	**Procured amount (g/10 MJ)**		**% Diff**	**Target (g/10 MJ)**
	**Advisory process**			
	**No (*n* = 269)**	**Yes (*n* = 87)**		
Bread and cereals^a^	329	326	-1%	280
Whole grain, g ingredient	115	112	-2%	
Potatoes	94	97	3%	100
Vegetables total^b^	253	273	8%	278
Fruit total^c^	180	188	-4%	277
Pulses, dry^d^	11	14	22%	30
Processed plant-based protein-rich foods^e^	2.7	2.4	-10%	-
Tree and ground nuts	1.2	2.0	59%	15
Seeds^f^	3.5	4.6	33%	9
Milk	254	263	3%	364
Dairy foods^g^	51	56	10%	36
Cheese	20	19	-6%	20
Plant-based dairy alternatives	9	8	-12%	-
Meat total^h^	58	48	-19%	47
Beef and lamb	18	10	-45%	9.5
Pork	11	12	8%	9.5
Poultry	29	26	-12%	28
Egg	20	24	20%	23
Fish, total^h^	53	50	-5%	63
Fats, plant-based^i^	45	53	18%	46
Fats, animal-based^i^	25	16	-37%	4
Coffee and tea	3	3	3%	3
Discretionary foods and beverages	28	30	7%	26
Condiments and seasoning	16	17	7%	15

^a^Combination of grains/flour and bread; ^b^Includes mushrooms; ^c^Includes berries, dried fruit, and a limited amount of fruit juice; ^d^Pulses purchased as a mix of cooked and dry pulses but are here expressed as dry weight ^e^Contains different soy-, pea-, and mycoprotein-based products (tofu, patties etc.); ^f^Does not include seeds in bread; ^g^All other dairy products except milk, cheese and butter. The target for the sum of milk and dairy foods should be 400 g/10 MJ; ^h^Meat and fish are predominantly raw but also contain limited amounts of processed products; ^i^Includes fat-based products, e.g., sauces and dressings

**Table 4:** Content of nutrients and carbon footprint of food procurement in 2022 in childcare centers who have and have not received advisory process (per 10 MJ), % difference between the two groups and comparison to recommendations. Nutrients not fulfilling recommendations are marked in bold

**Nutrient**^**a**^	**Nutrient content/carbon footprint per 10 MJ**		**% Diff**	**Recommendation **^**b**^
	**Advisory process**			
	**No (*n* = 269)**	**Yes (*n* = 87)**		
Protein, total, E%	**13**	**13**	-1%	10–20 (15)
Carbohydrates, E%	52	52	1%	45–60 (52–53)
Added sugar, E%	3	3	6%	≤ 10
Fat, total, E%	**35**	**35**	-1%	25–40 (32–33)
Saturated fatty acids, E%	**12**	**12**	-4%	≤ 10
n-3 fatty acids, E%	1.4	1.4	2%	≥ 1
Dietary fiber, g	38	39	2%	≥ 20
Vitamin A, RE	1160	1168	1%	≥ 660
Vitamin D, µg	**3**	**3**	-4%	≥ 19
Vitamin E, α -TE	15	17	12%	≥ 9
Thiamine, mg	1.5	1.5	2%	≥ 1.1
Riboflavin, mg	1.5	1.5	3%	≥ 1.3
Niacin, NE	24	24	-1%	≥ 17
Vitamin B6, mg	1.8	1.8	0%	≥ 1.3
Folate, µg	487	503	3%	≥ 151
Vitamin B12, µg	5.4	5.1	-5%	≥ 1.5
Vitamin C, mg	151	153	2%	≥ 57
Sodium, mg	**2730**	**2758**	1%	≤ 2400^c^
Potassium, mg	**3363**	3439	2%	≥ 3396
Calcium, mg^d^	**820**	**847**	3%	≥ 1132
Magnesium, mg	369	381	3%	≥ 226
Phosphorus, mg	1547	1571	2%	≥ 887
Iron, mg	**13**	**13**	1%	≥ 15
Zinc, mg	11	11	-1%	≥ 11
Iodine, µg	171	170	-1%	≥ 170
Selenium, µg	48	48	-1%	≥ 47
**Carbon footprint**^**e**^				
Metric 2, kg CO_2_e	3.9	3.6	-9%	
Metric 2 + 4, kg CO_2_e	15.4	13.2	-14%	13.1^f^

^a^Bioavailability and losses of vitamins and minerals from cooking not accounted for. ^b^Based on recommended nutrient density per 10 MJ for dietary planning for 2–5-year-olds from the Nordic Nutrition Recommendations 2012. Macronutrient recommendations are expressed as acceptable ranges for individuals and narrower ranges for planning purposes in parentheses. ^c^Based on recommendation for adults. ^d^An estimated 136 mg of calcium per 10 MJ can be added from 1.13 l of water, bringing the total amount of calcium to 983 mg/10 MJ in childcare centers that have not gone through advisory process and 956 mg/10 MJ in childcare centers that have. ^e^Based on Cool Food Pledge metrics [7]. ^f^Based on 25% reduction target from common 2018 baseline

## Discussion

This study analyzed changes in dietary composition and CF of food procurement in childcare centers in the City of Copenhagen from 2018 to 2022, following a new food strategy in 2019. A 15% decrease in total food-related GHGE was observed, primarily driven by decreased procurement of ruminant meat. The nutrient composition was not substantially changed, with protein, calcium, iron, and vitamin D below recommendations, and total and saturated fat and sodium above recommendations in both measurement years.

The observed CF reduction is in line with WRI’s analysis of the CFP signatories’ collective progress through 2022 compared to their respective base years (2015–2018) [35]. This analysis shows an overall 10% per-plate GHGE reduction across sectors (cities, companies, health care, restaurants and universities), with the "City" sector achieving a greater reduction of 24%. Similar to the present study, a large-scale study in the Oakland Unified School District, U.S, analyzed the environmental impact of the school district's food procurement changes over two years, finding a 14% reduction in CF by decreasing animal-based products and increasing plant-based and sustainable meat options [36]. Nutritional outcomes were not evaluated in either of these studies.

In order to reach the 25% CF reduction goal before 2025 as outlined in Copenhagen municipality’s food strategy and simultaneously improve nutritional quality, continued efforts to decrease meat consumption and animal-based fats should be made. This should be paired with substantial increases in especially consumption of pulses, nuts, and seeds, which were only moderately increased from the 2018 baseline and far below target amounts. Increases in these foods would contribute to increasing iron and protein content and protein quality, while decreased use of animal-based fats would bring down total and saturated fat content [23]. In both Finland and Norway, recent comparisons of young children’s dietary intake with the EAT Lancet reference diet have revealed substantially lower intakes of especially legumes and nuts and higher intakes of red meat and dairy compared to the reference diet [17, 37]. An optimization study of school meals in France showed that the best trade-off for decreasing the environmental impacts of school meals without compromising nutritional quality, was increasing the number of vegetarian meals and serving non-ruminant meat and fish with other meals [15]. Similarly, a case study on school meals in Copenhagen found that implementing scenarios representing meals with no beef, meals following food-based dietary guidelines, or vegetarian meals could achieve sizable GHGE reductions of 32–64%. However, nutritional outcomes were not evaluated [38]. In the present study, milk consumption, although somewhat interchangeable with the higher-than-target consumption of other dairy foods, was below target and should not be further decreased—rather increased—due to calcium and the total amount of milk and other dairy products being below recommendations. Childcare centers in Denmark are recommended to serve one small glass of milk per day per child in addition to dairy products used in cooking [39].

In June of 2023, new Nordic Nutrition Recommendations were published [40], which saw the recommended amounts for several nutrients increase from the previous recommendations (in children, most notably vitamin E, folate, vitamin B12 and magnesium). In the present study, the new recommendations are non-consequential for most nutrients since the observed amounts of the aforementioned nutrients exceed recommendations by a margin. In the case of calcium, the new recommendations correspond to approximately to 970 mg/10 MJ for 1–3-year-olds and 1250 mg/10 MJ for 4–6-year-olds, still emphasizing the importance of focusing on this nutrient. The new recommendations for iron on the other hand are fulfilled for 4–6-year-olds but not for 1–3-year-olds with the presently observed 13 mg/10 MJ.

Several different factors may have contributed to the observed changes in CF and food procurement patterns in the present study. Implementation of the 2019 food strategy in Copenhagen builds on previous experiences from extensive efforts to increase the share of organic foods within public food service. These efforts have already been at play for more than two decades in Copenhagen and have included upskilling efforts among food professionals, procurement monitoring, and an organic cuisine labeling initiative [41, 42]. Such efforts may have shifted food procurement patterns ahead of the 2019 food strategy by e.g. increasing procurement of fruit and vegetables [43], making further changes harder to achieve, but at the same time, both food professionals and consultants can draw on past experience in the implementation of the new strategy. A change in the political discourse and wider societal awareness and urgency of the climate crises might also have contributed to shifting procurement patterns independently of the new food strategy and the initiatives it entailed. Irrespective of the causes behind the observed changes, the development can be considered positive with regard to climate impact and, if continued, would see the municipality reach its target of 25% CF reduction from food service by 2025, given that other public food service settings also achieve their reduction targets. However, continued monitoring of nutritional quality is important to ensure synergistic benefits to environmental and health aspects of food service [22].

Despite the municipality’s childcare centers being on a good trajectory to reaching the reduction target, the next few years will require an intensive effort to fully achieve set targets. The institutions that have taken the lead in the transition may be the ones with the most capacity to implement changes (so-called “early adaptors”) and getting the remaining institutions to reach their potential might be more challenging. Some of the observed changes may also be considered as “low-hanging fruit” in the transition, e.g., an initial steep reduction in ruminant meat, which yields a large CF reduction. Further improvements, perhaps more difficult ones, will need to be made (e.g., the integration of more plant-based protein sources in all meals of the day). A continued advisory effort will likely be paramount in seeing the project to a positive outcome of reaching CF reduction and improved nutritional quality. The food procurement of institutions that had gone through an advisory process was, in general, more in line with the guidelines (e.g., targets were reached for CF reduction and ruminant meat procurement), indicating that advisory efforts may help in facilitating a transition or ensuring that the transition happens in a nutritionally correct way. However, to meet guidelines, further improvements in nutritional quality should also be made in institutions that have received an advisory process, and therefore, future efforts should have a heightened focus on increasing plant-based protein sources. These efforts will be even more important in the years to come since the municipality has decided that ruminant meat should no longer be served in childcare centers and schools from the summer of 2024 [44].

Several strengths and limitations apply to the presently used study design and data. The procurement data used for analyses allow for detailed and comprehensive analyses with near-complete representativeness of the municipal childcare centers, low participant burden, and low level of subjectivity (minimal social desirability bias). Due to the uncontrolled nature of this study, the observed changes cannot be attributed to specific initiatives of the new food strategy. However, this study has the strength of representing a quasi-experiment in a real-world setting and offers quantitative monitoring of temporal trends in both CF and diet composition of food procurement. Therefore, the case of Copenhagen can serve as a real-life example to inspire and inform future public food service actions elsewhere.

This study looked at the childcare sector in the municipality as a single unit in a pooled analysis, but there might be substantial inter-institutional variation, which has not been accounted for and should be studied further. Such analyses require the municipality to be able to ensure procurement data of sufficient quality on institutional level.

Uncertainties apply to the procurement, nutritional, and CF data, which warrant caution in the interpretation of the results. The procurement data does not cover the entirety of the food procurement in the childcare centers since suppliers providing limited amounts of a variety of foods were not included. Therefore, complete representativeness across food groups cannot be guaranteed. The CFP calculator does not allow for country-specific CF calculations and contains relatively few food commodities, making it necessary to make assumptions and simplifications in the pairing with food composition data. Furthermore, the CFP calculator uses retail weight as the input unit of foods for estimation of CF, except meat, for which boneless weight should be used [7]. In the present study, the edible weight was used for all foods, resulting in a mismatch between the functional unit of fruit and vegetables and a consequent underestimation of the CF of these foods. Importantly, however, the same methodology was followed for both measurement years, and therefore, results are comparable across time despite absolute estimates being less reliable. Finally, more environmental footprints should be considered for a more comprehensive evaluation of the environmental consequences of the observed shifts in food procurement.

## Conclusions

In conclusion, the findings of this study demonstrate that a meaningful reduction in the environmental impact of food service within childcare centers can be achieved while largely maintaining nutritional quality. The study also highlights the importance of monitoring the nutritional quality of public food service as efforts to decrease CF are made, since CF reduction strategies in food service do not guarantee improved nutritional quality. To ensure the nutritional quality of the food service at childcare centers, future efforts should focus on not only further decreasing the consumption of meat and animal-based fats but also increasing the use of plant-based protein sources (pulses, nuts, and seeds) and ensuring calcium supply through sufficient dairy products. Providing training to food-service providers could be important in these efforts.

By providing insights from a real-world setting, this study can inspire others in the formulation and implementation of future sustainable food service strategies. When done correctly, sustainable public food procurement and catering can improve two sustainability dimensions simultaneously (environment and nutrition) and will be important in achieving a wider shift to a more sustainable food system.

## Data Availability

The data that support the findings of this study are available from the City of Copenhagen, but restrictions apply to the availability of these data, which were used under license for the current study and so are not publicly available. Data are, however, available from the authors upon reasonable request and with permission of the City of Copenhagen’s Budget Team in the Children and Youth Administration.

## Abbreviations

CF: Carbon footprint
CFP: Cool Food Pledge
CO_2_e: Carbon dioxide equivalent
GHGE: Greenhouse gas emissions
WRI: World Resources Institute
